# Predictors of Mental Health Status among Quarantined COVID-19 Patients in Saudi Arabia

**DOI:** 10.3390/healthcare9101271

**Published:** 2021-09-26

**Authors:** Abdulaziz A. Alodhayani, Khalid M. Almutairi, Fahda A. Alshobaili, Adel F. Alotaibi, Ghadah Alkhaldi, Jason M. Vinluan, Hadeel Mohammed Albedewi, Lamis Al-Sayyari

**Affiliations:** 1 Department of Family and Community Medicine, College of Medicine, King Saud University, Medical City, Riyadh 11362, Saudi Arabia; falshobaili@ksu.edu.sa; 2 College of Applied Medical Sciences, King Saud University, Riyadh 11433, Saudi Arabia; kalmutairim@ksu.edu.sa (K.M.A.); ghalkhaldi@ksu.edu.sa (G.A.); jvinluan@ksu.edu.sa (J.M.V.); hadeelbed@gmail.com (H.M.A.); lalsayyari@ksu.edu.sa (L.A.-S.); 3 Ministry of Health, Riyadh 12628, Saudi Arabia; alotaibiadel18@gmail.com

**Keywords:** COVID-19, mental well-being, quarantine, coping mechanism, elderly, Arab

## Abstract

Background: The negative psychological impact of COVID-19 in the general population has been well documented. Similar studies among those who were infected and who underwent quarantine remain scarce, particularly in the Arab region. The present study aims to fill this gap. Methods: In this cross-sectional study, suspected/confirmed COVID-19 individuals who were quarantined in the Ministry of Health (MOH) facilities were invited to participate in an online survey. All consenting participants answered a generalized questionnaire that included demographic characteristics, as well as a five-part questionnaire that assessed the symptoms of depression, anxiety, insomnia, and distress. Results: A total of 335 suspected/confirmed COVID-19 individuals (198 males and 137 females) participated. Being female is associated with increased risk of depression (odds ratio OR 1.8 (confidence interval, CI 1.1–3.1; *p* = 0.03)) as well as being employed by the government (OR 2.8 (CI 1.1–7.0; *p* = 0.03)). Level of education (OR 2.3 (CI 1.0–5.4; *p* = 0.049)) and employment in government (OR 3.0 (CI 1.2–7.8; *p* = 0.02)) were significantly associated with distress. Increasing age (45 years and above) appeared to be protective against distress (OR 0.2 (CI 0.02–0.69; *p* = 0.008)), as well anxiety and sleep pattern (OR 0.3 *p* < 0.05). Conclusion: Findings of the present study highlight that infected COVID-19 populations are at higher risk for acute and detrimental psychological well-being during quarantine and/or self-isolation. Identification of the coping mechanisms of older adults during periods of distress may prove beneficial in the pandemic preparedness of younger generations.

## 1. Introduction

More than a year has passed since the coronavirus disease-19 (COVID-19) pandemic began. Globally, confirmed COVID-19 cases have surpassed 200 million, including more than 4.2 million deaths [1]. Given this catastrophic viral outbreak of historic proportions, the general population experienced a consequential increase in acute psychological distress [2]. Factors that have exacerbated the substantial decrease in the general public’s mental health status include stringent lockdowns and self-isolation to minimize the number of infected cases [3]. Moreover, the sense of uncertainty and fear towards the COVID-19 as a threat to one’s way of life has been challenging [4]. This added psychological stress may aggravate existing psychiatric disorders or may aggravate its symptoms [5]. In fact, new-onset mental issues like psychosis and affective disorder were diagnosed in about one-third of British COVID-19 patients in the beginning of the pandemic [6]. A recent systematic review suggested that quarantine is associated with harmful psychological and physical effects [7,8]. These aforementioned effects have been observed to linger long after the disease is controlled, as observed in previous pandemics such as the severe acute respiratory syndrome-coronavirus 1 (SARS-CoV-1) [9]. In a recent Irish National Survey, COVID-19-related quarantines were associated with bouts of anxiety and depressive symptoms [10]. Similarly, in Southwest China, the prevalence of anxiety and depression was twice as much in quarantined participants than those who were not [11]. 

In Saudi Arabia, initial studies on COVID-19 were mainly retrospective and geared on the identification of risk factors for COVID-19 infection and mortality [12,13,14,15], with some studies focused on lifestyle changes and treatment [16,17]. However, studies on mental health are still limited. A recent observation by Alkhamees and colleagues showed that about 25% of the Saudi population experienced moderate to severe psychological effects during the pandemic [18]. Moreover, within the Saudi demographics, subpopulations such as those from the academia and healthcare students experienced acute mental health disorders that were commonly reported [19,20]. To date, there is a scarcity of evidence on the mental health status of locals with suspected or confirmed SARS-CoV-2 infection who were quarantined. The present study aims to fill this gap.

## 2. Materials and Methods

### 2.1. Study Population 

**Figure 1:**
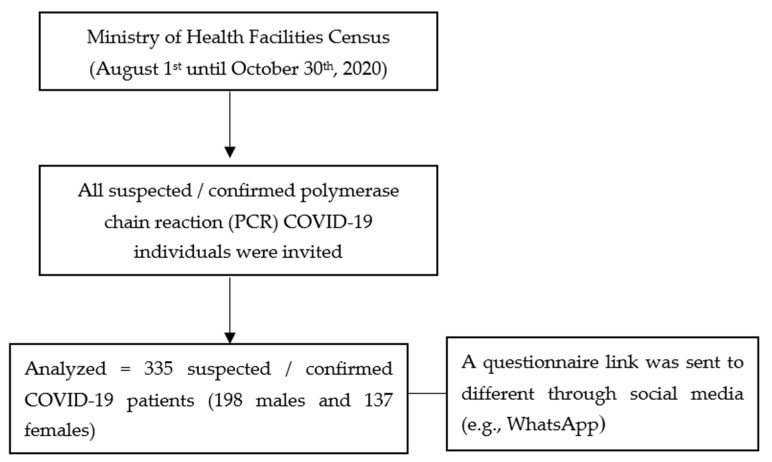
Flowchart of participants.

In this cross-sectional study, suspected or polymerase chain reaction (PCR)-confirmed COVID-19 individuals residing in Saudi Arabia who were quarantined in the Ministry of Health (MOH) facilities were invited to participate in this study from 1 August until 30 October 2020. Ethical clearance was obtained from the Institutional Review Board (IRB) of King Fahad Medical City (KFMC) (IRB Log No. 20-397E, approved 22 June 2020), Riyadh, Saudi Arabia prior to data gathering. To minimize risk of infection, data were collected using an online survey. A questionnaire link was sent to different suspected or confirmed COVID-19 individuals through social media. Consenting participants were sent regular reminders every 3 days during the study period. Figure 1 presents the flow diagram of the participants. 

### 2.2. Instruments 

All participants answered a 5-part questionnaire that assessed the symptoms of depression, anxiety, insomnia, and distress. The first part of the questionnaire was composed of the demographic characteristics, which included age, sex, marital status, level of education, and employment status. The second part was the 9-item Patient Health Questionnaire (PHQ-9; range, 0–27) [21], while the 7-item Generalized Anxiety Disorder (GAD-7) scale (range, 0–21) was used to identify the anxiety level among healthcare providers [22]. All scores were calculated and interpreted using a scoring manual from previous studies [21,22,23,24]. For PHQ-9, a total score of 15–21 was considered as with severe depression, 0–4 as normal, 5–9 as mild, and 10–14 as having moderate depression. With regards to GAD-7 anxiety, a score of 15–21 was considered as severe anxiety, 0–4 as normal, 5–9 as mild, and 10–14 as moderate anxiety. The 7-item Insomnia Severity Index (ISI; range, 0–28) was used to assess and categorized ISI into the following categories: normal (0–7), subthreshold (8–14), moderate (15–21), and severe (22–28) insomnia [23]. The last part of the questionnaire was the 22-item Impact of Event Scale–Revised (IES-R; range, 0–88) and was recorded as normal (0–8), mild (9–25), moderate (26–43), and severe (44–88) distress [24].

### 2.3. Statistical Analysis

Data were analyzed using SPSS windows v. 22 (Chicago, IL, USA). Categorical data were presented as frequencies and percentages (%) while continuous data were presented as mean ± standard deviation. The Chi-square test was used to determine associations and differences within variables of interest. Logistic regression analysis was done to determine significant predictors. Significance was set at *p* < 0.05.

## 3. Results

A total of 335 suspected/confirmed COVID-19 patients (198 males and 137 females) participated in the study. The demographic characteristics of the participants are shown in Table 1. The majority of the respondents (*n* = 310, 92.5%) were Saudis and were mostly living in Riyadh (*n* = 257, 76.7%). The mean age was 33.7 ± 9.7 years. More than half the respondents were married (*n* = 194, 57.9%), had at least a bachelor’s degree (*n* = 190, 56.7%), and employed by the government (*n* = 174, 51.9%). The mean duration of quarantine was 14.3 ± 7.3 days.

The mental health status of respondents as assessed using PHQ-9, GAD-7, ISI, and IES-R are shown in Table 2. Under the PHQ-9, the prevalence of respondents having mild to severe depression was 63.6%, of which 39 (11.6%) fell under the severe category. Using GAD-7, 49 respondents (14.7%) fell under the moderate to severe category of anxiety. Under the ISI, 57.9% of respondents claimed to normal sleep pattern whereas 14.6% had moderate to severe insomnia. Lastly and according to IES-R, more than two-thirds (68.6%) had some level of distress, the majority of whom were mild (40.3%), 17.6% were moderate, and 10.7% were severe.

The mental health status of male and female respondents was compared in Table 3 and showed no significant differences in PHQ-0, ISI, and IES-R. Under GAD-7, however, the prevalence of mild to severe anxiety was significantly higher in females than males (44.5% vs. 33.8%; *p* = 0.048). 

Demographic characteristics that may affect the level of depression are shown in Table 4. There was a significant association between nationality and depression, with non-Saudis being more likely to have some level of depression than Saudis (*p* < 0.001). Likewise, mild to severe depression were more commonly observed among government employees and those earning more than 5000 SAR monthly (*p*-values 0.01 and 0.02, respectively).

Lastly, the predictors of mental health were determined using logistic regression models (Table 5). Being female (odds ratio OR 1.8 (confidence interval, CI 1.1–3.1; *p* = 0.03)) and a government employee (OR 2.8 (CI 1.1–7.0; *p* = 0.03)) were significant predictors of depression. As for distress, education (OR 2.3 (CI 1.0–5.4; *p* = 0.049)) and employment in government (OR 3.0 (CI 1.2–7.8; *p* = 0.02)) were significant positive predictors. Increasing age, particularly among those aged 45 and above, was observed to be protective against distress (OR 0.2 (CI 0.02–0.69; *p* = 0.008)), as well anxiety and sleep patterns (OR 0.3 *p* < 0.05) for ages 35 to 54 (not included in table).

## 4. Discussion

The present study observed that majority of suspected/confirmed COVID-19 cases had depression and distress at varying degrees during isolation, accompanied by a high prevalence of insomnia and anxiety. Predictors affecting negative mental health status in the studied population include the female sex, being employed by the government, and level of education, while increasing age appears to confer protection against distress. The present findings are arguably the first to identify demographic characteristics of COVID-19 patients serving quarantine period who are at high risk for mental health issues in the Arabian population.

Pandemics and outbreaks in general elicit collective fear from the public and social stigmatization among infected individuals due to inherent clinical uncertainties, more so if the spread of illness and death are substantial [25,26]. Although a quarantine period is a time-tested strategy to control the spread of infection, it has also been known to elicit harmful psychological impacts among complying people, even in the absence of a pandemic, since individuals are forced to isolate themselves from the society and abruptly change their way of life for the collective good [7,27]. Many large-scale observational studies have also pointed out the negative consequences of quarantine and isolation measures during COVID-19 in both the general and vulnerable population [28,29,30,31], as well as its direct association with the severity of COVID-19 restriction [32]. These previous observations are also reflected in the present study.

In the present study, being a female increases predisposition to depression among quarantined COVID-19 patients. This finding agrees with previous studies done among Arab populations where acute depression and anxiety were significantly more common in women than men during the pandemic [33,34]. Women in Middle Eastern cultures carry the burden of the family’s overall wellness, and this pressure to deliver is heightened if the woman is employed as she needs to balance career and family [35]. Therefore, being infected with COVID-19 and having to quarantine to recover may increase the psychological stress load on top of the societal responsibility already imposed on an Arab woman [36]. The higher prevalence of depression and anxiety among females may also arise from the stigma associated with being infected, not to mention being the vulnerable sex when it comes to domestic violence, a common occurrence in the Arab region during the pandemic [37].

Another interesting finding in the study is the protective effects of increasing age against negative mental health outcomes. Despite being the most vulnerable group to SARS-CoV-2 infection and COVID-19 mortality, older adults as a subpopulation have been observed to be much more resilient to the anxiety, depression, and stress-related mental health disorders compared to their younger counterparts in the ongoing COVID-19 pandemic [38]. Older adults tend to be more internally resilient, which means they have developed a sense of internal control to adapt to changes brought about by external threats and perceived crisis, better known as the need–threat internal resiliency theory [39]. Another explanation is the adaptive coping of older adults, who tend to be more positive [40]. Although not assessed in the present study, there is strong evidence of the elderly staying pro-active in times of crises, with coping mechanisms that include maintaining a daily routine, seeking social support, and maintaining a positive mindset to buffer the negative effects of stressors [40,41]. 

Lastly, the present study found that COVID-19 patients who were non-Saudis had a higher tendency for depression as compared to Saudis. The prevalence of mental health issues among select expatriates in the Middle East during the COVID-19 pandemic were observed to be high in some Arab nations [42,43]. In some subpopulations, however, such as those working in the healthcare sector, where the majority of workers are expatriates, levels of mental stress appear to be on the lower proportion as compared to the general population [44]. Among the possible reasons that can aggravate negative psychological well-being among expatriates include the severity of the pandemic in their home nations, where it was observed to be associated with levels of depression and post-traumatic stress disorder among foreigners employed in the United Arab Emirates [45]. 

The authors acknowledge several limitations. First, the study was conducted utilizing a cross-sectional design that limits determining causal conclusions. Second, the sample size was not large enough and thus cannot be generalized and applies only to quarantined and suspected/confirmed COVID-19 patients. However, the lack of comparison with non-quarantined participants is justified, as there is already a plethora of evidence concerning the psychological well-being of these subpopulations as compared to the cohort used in the study, which remained under-investigated at the time of writing. Third, only a handful of non-Saudis participated during data collection, creating a big discrepancy in sample size, which limits meaningful comparisons between the Saudi participants. In addition, data were collected during the first wave of the pandemic, at a time when institutional quarantines for infected patients were implemented in Saudi Arabia. Lastly, the findings of the strength of the relationships between predictors and mental health are rather small, which may capture only the probable negative psychological impact of COVID-19 isolation. However, the study highlighted valuable information that may prevent the manifestation and severity of psychological disorders. The findings may help healthcare administrators and policymakers to formulate strategic plans to alleviate the risk of depression and mental distress based on the identified vulnerable subjects. Further studies are needed exploring the mental health outcomes of recovered and quarantined patients in the latter stage of the pandemic. In addition, future studies are needed to explore interventions or coping strategies that will help patients to overcome or maintain mental health in different unprecedented situations such as the COVID-19 pandemic. As such, the findings may only apply during the initial stages of the outbreak, as coping and adaptive mechanisms may have already been developed in the general public mental health as more knowledge about the pandemic is acquired over time. 

## 5. Conclusions

In summary, the majority of the quarantined patients in Saudi Arabia with suspected/confirmed COVID-19 experienced varying levels of depression and mental distress. Predictors of mental health issue severity include being female, being employed by the government, and having higher levels of education. Increasing age appeared to be protective against the negative psychological impact of COVID-19 isolation. Findings of the present study identified possible subpopulations at risk who may require additional services that cater to mental well-being while serving the mandatory self-isolation during infection. Additional studies among older adults may offer insights with respect to coping mechanisms and adaptive strategies that can ultimately be used in the general population’s pandemic readiness in future outbreaks.

## Figures and Tables

**Table 1:** Demographic characteristics of participants.

Parameter	
*n*	335
Age (years)	33.7 ± 9.7
Sex	
Male	198 (59.1)
Female	137 (40.9)
Nationality	
Saudi	310 (92.5)
Non-Saudi	25 (7.5)
Marital Status	
Single	128 (38.2)
Married	194 (57.9)
Divorced or Widowed	13 (3.9)
Level of Education	
Highschool and Below	60 (17.9)
Bachelor	190 (56.7)
Postgraduate	85 (25.4)
Employment Status	
Student	74 (22.1)
Unemployed	36 (10.7)
Private	51 (15.2)
Government	174 (51.9)
Residence	
Riyadh	257 (76.7)
Outside Riyadh	78 (23.3)
Monthly Income (SAR)	
Less than 5000	79 (29)
5000–10,000	105 (31.3)
Above 10,000	133 (39.7)
Quarantine duration	14.3 ± 7.3

Note: Data are presented as mean ± SD for continuous variables and frequencies (%) for categorical variables.

**Table 2:** Mental health status of participants according to different scales used.

Scale	
**PHQ-9**	
Minimal	122 (36.4)
Mild	101 (30.1)
Moderate	73 (21.8)
Severe	39 (11.6)
**GAD-7**	
Minimal	207 (61.8)
Mild	79 (23.6)
Moderate	29 (8.7)
Severe	20 (6)
**ISI**	
Normal	194 (57.9)
Subthreshold	92 (27.5)
Moderate	36 (10.7)
Severe	13 (3.9)
**IES-R**	
Normal	105 (31.3)
Mild	135 (40.3)
Moderate	59 (17.6)
Severe	36 (10.7)

**Note**: Data are presented as frequencies (%); PHQ-9: 9-item Patient Health Questionnaire. GAD-7: 7-item Generalized Anxiety Disorder scale. ISI: Insomnia Severity Index. IES-R: Impact of Event Scale–Revised.

**Table 3:** Differences in mental health status according to sex.

	Male	Female	*p* Value
	*n* = 198	*n* = 137	
PHQ-9			0.11
Normal	79 (39.9)	43 (31.4)	
Mild to severe	119 (60.1)	94 (68.6)	
GAD-7			0.048
Normal	131 (66.2)	76 (55.5)	
Mild to severe	67 (33.8)	61 (44.5)	
ISI			0.15
Normal	121 (61.1)	73 (53.3)	
Mild to severe	77 (38.9)	64 (46.7)	
IES-R			0.34
Normal	66 (33.3)	39 (28.5)	
Mild to severe	132 (66.7)	98 (715)	

**Note**: Data are presented as frequencies (%); Pearson Chi-square was used to determine association between psychological characteristics and gender. PHQ-9: 9-item Patient Health Questionnaire. GAD-7: 7-item Generalized Anxiety Disorder scale. ISI: Insomnia Severity Index. IES-R: Impact of Event Scale–Revised.

**Table 4:** The association between demographic characteristics and depression.

	Normal	Mild to Severe	*p-*Value
	*n* = 122	*n* = 213	
Nationality			<0.001
Saudi	121	189	
Non-Saudi	1	24	
Marital Status			0.6
Single	49	79	
Married	67	127	
Divorced or Widowed	6	7	
Level of Education			0.54
Highschool and Below	22	38	
Bachelor	65	125	
Postgraduate	35	50	
Employment Status			0.01
Student	34	40	
Unemployed	18	18	
Private	21	30	
Government	49	125	
Monthly Income (SAR)			0.02
Less than 5000	45	52	
5000–10,000	29	76	
Above 10,000	48	85	

**Note**: Data are presented as frequencies; Pearson Chi-square was used to determine the association between demographic characteristics and depression.

**Table 5:** Demographic predictors of depression and distress among COVID-19 patients.

	Depression			Distress		
	OR	95% CI	*p* Value	OR	95% CI	*p* Value
Sex			0.03			0.99
Male	1.0			1.0		
Female	1.8	1.05–3.05		1.0	0.58–1.73	
Age (years)			0.05			0.002
17–24	1.0			1.0		
25–34	1.4	0.62–3.18	0.42	0.9	0.40–2.17	0.86
35–44	0.9	0.32–2.69	0.88	0.4	0.15–1.33	0.15
45–54	0.4	0.12–1.38	0.15	0.2	0.05–0.65	0.008
≥55	2.4	0.41–13.5	0.34	0.13	0.02–0.69	0.02
Marital Status			0.39			0.2
Single	1.0			1.0		
Married	0.9	0.49–1.95	0.95	1.4	0.66–2.81	0.40
Divorced/Widowed	0.4	0.11–1.60	0.20	4.9	0.85–28.1	0.08
Level of Education			0.37			0.10
Highschool	1.0			1.0		
Bachelor	0.9	0.52–1.89	0.97	1.2	0.64–2.34	0.55
Postgraduate	0.7	0.30–1.41	0.28	2.3	1.03–5.35	0.049
Employment Status			0.01			0.12
Student	1.0			1.0		
Unemployed	0.6	0.25–1.67	0.37	2.8	0.93–7.96	0.07
Private	1.2	0.50–3.04	0.65	2.4	0.91–6.47	0.08
Government	2.8	1.13–7.01	0.03	3.0	1.15–7.66	0.02
Monthly Income (SAR)			0.53			0.67
<5000	1.0			1.0		
5000–10,000	1.3	0.66–2.77	0.42	0.7	0.34–1.55	0.41
>10,000	0.9	0.44–2.13	0.93	0.9	0.37–2.05	0.76

**Note**: Binary logistic regression was used to determine the predictors of depression and distress. OR: Odds ratio. CI: Confidence interval. Significant at *p* < 0.05.

## Data Availability

All data are provided in the article.
